# Reimagining care: effectiveness of modifying an adolescent eating disorder intensive service

**DOI:** 10.1192/bjb.2024.45

**Published:** 2025-08

**Authors:** Ellen Hayes, Nicola Tweedy, Victoria Chapman

**Affiliations:** Specialist Child and Adolescent Eating Disorder Service, Royal Free Hospital, London, UK

**Keywords:** Feeding or eating disorders, anorexia nervosa, intensive eating disorder treatment, child and adolescent mental health services, adolescent eating disorders

## Abstract

**Aims and method:**

The COVID-19 pandemic prompted a surge in adolescent eating disorders and rapid changes in the delivery of intensive community treatments. This study investigates the modification from a group-based day programme to an intensive family treatment approach. A retrospective chart review was performed on data from 190 patients who accessed the intensive service for anorexia nervosa in the past 6 years. Outcomes from the traditional model were compared with the new intensive family model, namely length of admission, percentage median body mass index difference and transfers to in-patient services.

**Results:**

There was a significant reduction in the length of intensive treatment (from 143.19 to 97.20 days). The number of transfers to specialist eating disorder in-patient services also significantly reduced, and is decreasing year on year.

**Clinical implications:**

The findings hold particular relevance as intensive services for adolescent eating disorders continue to be established within health services, with no clear unified approach to treatment.

Eating disorders have one of the highest mortality rates of all psychiatric disorders, and are particularly harmful in children and adolescents because they affect normal growth and development.^1^ Some adolescents with anorexia nervosa will require in-patient or more intensive care for medical or psychological stabilisation over the course of their illness. The rates of this varies across countries and health services, with the Maudsley Centre for Child and Adolescent Eating Disorders reporting that 27% of patients with anorexia nervosa required admission to their day programme and/or an in-patient service.^2^ Although often necessary, the benefits of in-patient care for adolescents beyond medical stabilisation is disputed, as the risk for readmission and relapse is high.^3^ Alternative community intensive treatments can offer higher levels of care while allowing the young person to remain within their social, family and educational context. Although literature concerning this treatment increasing, there is still no clear unified approach to treatment.^4^

## Previous findings

In studies reporting on adolescent eating disorder intensive services, there are high variations in the approach that they take. There is variability in the amount and length of treatment that is offered and the expected outcomes. Most community intensive day programmes follow a family-based approach to treatment, but there are variations on whether a group-based aspect is also incorporated. Despite the variations, the findings indicate that community intensive programmes can improve physical and psychological outcomes for adolescents who are not advancing in out-patient treatment.^5–9^

An evidence-based agreement on the most important elements of intensive treatment in adolescent eating disorders is needed to foster treatment programmes that are most helpful to improve outcomes. This paper evaluates the effectiveness of a modification in treatment from a traditional group-based programme to an intensive family-based programme.

## Method

This service evaluation used a retrospective chart review with patients aged between 10 and 18 years old who had accessed the Royal Free Hospital Eating Disorder Intensive Service (EDIS) between March 2017 and April 2023. The sample was split into two groups: those who accessed the service from March 2017 to March 2020, when the service was run as a traditional group-based day programme (*n* = 86); and those who accessed the service from April 2020 to April 2023, when the service was modified as an intensive family-based programme following the COVID-19 pandemic (*n* = 104). Patients whose treatment overlapped during this time were not included (*n* = 14).

As this is a service evaluation, National Health Service ethical approval was not required. This was discussed with the research and development department at the Royal Free Hospital, and registered with the quality governance team.

### Programme description: the EDIS

The EDIS was set up as a pilot in 2013, with the aim of reducing the numbers admissions from our service to specialist eating disorder units. The intensive service was set up to provide a step up in care to young people with restrictive eating disorders by providing a day service that was fully integrated with the specialist out-patient service. Patients who were not progressing with out-patient treatment or at immediate risk of hospital admission were offered a day service programme at the same site as their out-patient care. Patients could attend daily, up to seven days a week, to receive meal support with other patients. There was an on-site school hub where patients received education in the morning and then attended treatment sessions in the afternoon. Therapeutic interventions included family treatment for anorexia nervosa (FT-AN), as recommended by the National Institute for Health and Care Excellence,^10^ and therapies targeting the young person's individual needs as identified by their formulation. Patients who became medically unstable or were at high risk of refeeding syndrome were admitted to the acute paediatric ward for medical stabilisation and refeeding. The intensive service also provided a step down in care to patients admitted to specialist eating disorder units or paediatric wards, so that they could return to the community.

In 2020, the COVID-19 pandemic necessitated a change to the EDIS's treatment model, as it was no longer possible for patients to eat together, attend the school at the service or receive group therapy treatments. The local paediatric ward was closed, and it was more difficult to support patients at acute physical risk. Furthermore, there was a 59% increase in the number of referrals to the service received in 2021–2022 compared with 2019–2020. It became important to develop a programme that was not only safe and effective, but also efficient enough to cope with the increase in demand.

The solution was to develop the intensive family-based programme and offer intensive individualised meal support to patients with their families or carers on site or online. The current model is informed by FT-AN, and patients are no longer treated in a group environment and the school has been closed. In its new form as an intensive family-based programme, the service runs from 08.00 to 20.00 h, 7 days a week. It is led by a nursing team and supported by therapeutic care workers and a family therapist. The team is supported by the multidisciplinary out-patient team, and receives regular input and supervision from family therapists and psychologists.

The treatment is focused on supporting parents and carers to provide meal support, through practical on-site and online guidance and psychoeducation. Parents and carers receive skills-based individual meal support coaching by a member of the EDIS team, to help them to gain confidence in supporting their young person with their eating disorder. Online support can also be offered to facilitate eating in the home environment. The frequency of the support generally reduces as the family and young person gain confidence with eating. Parents are also offered daily contact with the team via telephone. Parents are offered an online 4-week psychoeducation group, which is run by the out-patient team. This is an opportunity for parents and carers to gain a better understanding of eating disorders and learn key skills in supporting their child. It is also an opportunity for parents to connect with other families, and we have recently introduced a fifth in-person session for parents to meet in person and support each other. A key aspect of the intensive service is for the treatment to be integrated within the out-patient team, allowing for consistency in clinicians and continuity of care throughout the patient's treatment journey. As well as attending EDIS, families continue weekly FT-AN sessions with their out-patient team. Additional family therapy can also be provided by a family therapist within EDIS, if needed.

Each young person is also offered individual input through weekly key sessions. These focus on motivational support, anxiety management and distress tolerance. Individual sessions can be stepped up to cognitive–behaviour therapy for eating disorders (CBT-E) or individual psychotherapy, if necessary, offered by an out-patient clinician. Education is supported by a specialist teacher within the team, who can liaise with schools to encourage re-integration while in EDIS. Dietitians also work closely with EDIS, and meet with families to provide individualised meal plans, guidance on nutritional rehabilitation and support with nasogastric feeding. Additionally, an on-site, consultant-led paediatric clinic has been introduced to support the management of patients at physical risk, with the aim of reducing acute paediatric admissions. Patients who require refeeding and need short periods of nasogastric tube feeding can now be treated within the department and do not need hospital admission.

Importantly, the treatment plan of a patient in EDIS is carefully personalised to each patient, considering their individual formulation and progress through treatment. Some families may attend EDIS every day and be supported for all meals and snacks, whereas others may only need support for a few meals a week. Families may attend EDIS every day at the beginning of their admission, and this may taper down to once a week as they transition back to being supported by just their out-patient team. Table 1 further demonstrates how the service worked previously, and the key aspects of the change in approach with a rationale for the change.

**Table 1:** Comparison of the traditional model and intensive family model, with rationale

Traditional model	Intensive family model	Rationale
Open 08.00–20.00 h, 7 days a week.	Open 08.00–20.00 h, 7 days a week.	Families can be supported for all meals, and do not feel ‘stuck’ at the weekend or over holiday periods.
Meal support sessions are facilitated by nurses and TCWs in a group, without the family present.	Nurses and TCWs provide support to individual families to effectively encourage their young people to eat, on site or online.	Families gain confidence to transfer the skills they have gained effectively at home, outside of the EDIS.
Young people remain in a group with other patients throughout the day. Family attends only for FT-AN input.	Patients attend with their families for the purpose of meal support and key sessions. They remain separate from other EDIS patients.	Meal support continues to be family based, strengthening the FT-AN approach.
Young people are educated on site by specialist teachers in the morning.	Young people are supported by a specialist teacher to attend school while also attending the EDIS. Tuition can be provided when school attendance is not possible.	Remaining within the school and community means important connections with friends and teachers are maintained.
Individual therapies and FT-AN are provided to young people and families in the afternoon.	Young people attend weekly ‘key sessions’ facilitated by nurses and TCWs. Weekly FT-AN sessions are coordinated by the out-patient team.	Young people are offered individualised support that may be more accessible than individual therapies at a low weight, and connections with the out-patient team are maintained.
Young people are admitted to a paediatric ward if they are at risk of refeeding or need nasogastric feeding.	Medical monitoring for refeeding can be provided within the EDIS in partnership with paediatricians. Nasogastric feeding is provided by nurses within the EDIS.	Less burden is placed on paediatric wards, where managing eating disorders can be challenging. Lengthy ward and subsequent tier 4 admissions, which can negatively affect recovery, are more likely to be avoided.

TCW, therapeutic care worker; EDIS, Royal Free Hospital Eating Disorder Intensive Service; FT-AN, family treatment for anorexia nervosa.

### Selection criteria

The sample consisted of admissions of young people aged 10–18 years who had accessed EDIS from March 2017 to April 2023. All admissions were for young people with a diagnosis of anorexia nervosa according to the DSM-5. Admissions were excluded if there was insufficient or unclear data for analysis, or if admission dates overlapped across the two measurement periods.

### Measures

#### Physical outcomes

Weight gain was calculated as a percentage of median body mass index (%mBMI). The difference in %mBMI from admission to discharge from EDIS was measured. Because of inconsistencies in data being recorded on clinical records, there were several patients with missing data from this variable (*n* = 23 traditional model, *n* = 8 intensive family model). These patients were omitted from the analysis of this variable.

#### Process outcomes

Length of admission in days was calculated using the first and last day of EDIS admission. Readmission was counted when there was more than one admission for the same patient within 3 years. In-patient admissions were counted if a patient was admitted to a specialist eating disorder unit relating to their eating disorder during their EDIS treatment.

### Statistical analysis

Data was analysed using JASP software (Windows 64-bit version 0.17.3; see https://jasp-stats.org). Descriptive values for the age, gender, duration in EDIS and %mBMI of admissions were derived. For examining the length of admission, Shapiro–Wilk tests of normality indicated data were not normally distributed. Therefore, Mann–Whitney *U*-tests were carried out on these data. Independent samples *t*-test were used to examine differences in %mBMI between groups. A chi-squared test was used to determine differences in the number of transfers to specialist eating disorder in-patient units.

## Results

**Fig. 1:**
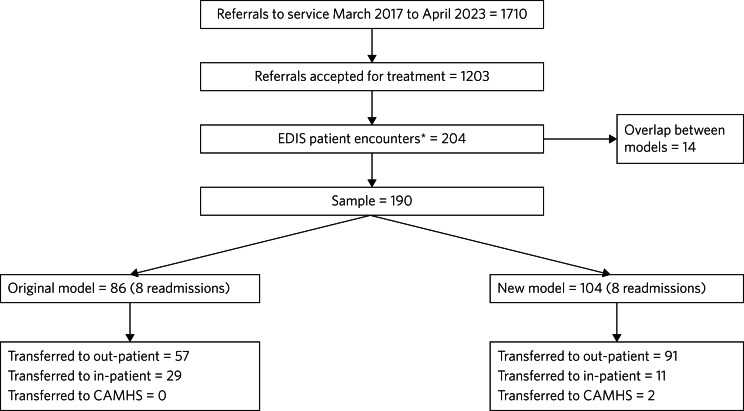
Flow diagram of EDIS patient encounters and care pathways on discharge. *Patients who were admitted to the EDIS from March 2017, and discharged by April 2023. CAMHS, child and adolescent mental health services; EDIS, Royal Free Hospital Eating Disorder Intensive Service.

A total of 190 participants who had used the EDIS from March 2017 to April 2023 were included in the study. The sample was separated into the traditional model group (March 2017 to March 2020) and intensive family model group (April 2020 to April 2023), reflecting the modification of our treatment approach during and after the COVID-19 pandemic. Any patient whose treatment overlapped during this time was not included (*n* = 14). Figure 1 demonstrates the number of referrals received and accepted into the EDIS in the past 6 years, how many accessed EDIS and if they were transferred to an in-patient service.

### Length of admission

As shown in Table 2, the median length of treatment was 135 days (interquartile range, 64–221 days) in the traditional model group, and 89 days (interquartile range, 59–137.75 days) in the intensive family model group.

**Table 2:** Patient characteristics split by traditional and intensive family models

	Group	*n*	Mean	s.d.	Range
Age	Traditional model	86	14.94	1.514	10–17
	Intensive family model	104	14.96	1.66	10–17
Gender	Traditional model	Female: 85 Male: 1	−	−	−
	Intensive family model	Female: 97 Male: 7	−	−	−
Duration in the EDIS	Traditional model	86	143.19 (median 135)	100.11	7–480^a^
	Intensive family model	104	97.20 (median 89)	53.43	8–220^a^
%mBMI at admission	Traditional model	63	79.73	8.91	61.67–104.3
	Intensive family model	96	80.89	8.77	63.05–106.8
%mBMI at discharge	Traditional model	63	88.37	9.82	70.0–106.6
	Intensive family model	96	89.08	7.68	63.28–103.4
%mBMI Difference	Traditional model	63	8.81	10.09	−4.9 to 35.37
	Intensive family model	96	8.19	7.50	−14.8 to 31.5

Sample sizes vary across variables because of missing data. EDIS, Royal Free Hospital Eating Disorder Intensive Service; %mBMI, percentage median body mass index.

a. Days in the EDIS from admission date to discharge date.

A Mann–Whitney *U*-test was performed to test whether the duration in EDIS differed significantly between groups. As demonstrated in Table 3, the results indicated that there was a significant difference in the duration in EDIS between each group (*U* = 5559.00, *P* < 0.01).

**Table 3:** *t*-Test analyses for transfers to in-patient services, length of admission and percentage median body mass index difference

Variable	Group	*n*	Test statistic
Transfer to in-patient services	Traditional model	86	*χ*^2^(1) = 6.03, *P* = 0.014, Cramer's *V* = 0.2
	Intensive family model	102	
Length of admission	Traditional model	86	*U* = 5559, *P* = 0.004, 95% CI 0.082–0.391
	Intensive family model	104	
%mBMI difference	Traditional model	63	*t*(157) = 0.444, *P* = 0.658, 95% CI –0.246 to 0.390
	Intensive family model	96	

Sample sizes vary across variables because of missing data. %mBMI, percentage median body mass index.

### Transfer to in-patient services

**Fig. 2:**
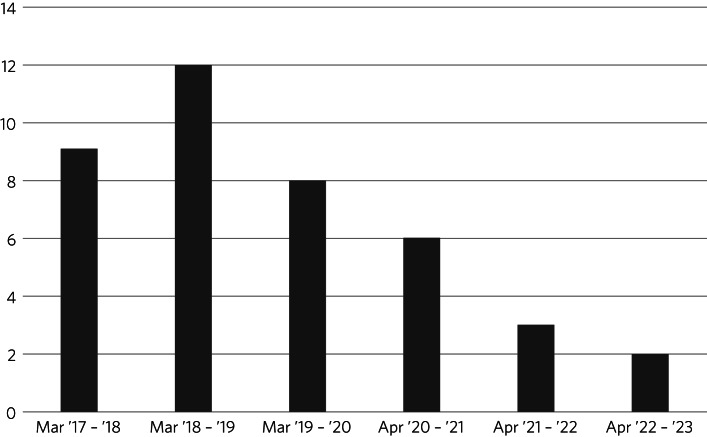
The number of admissions to a specialist eating disorder in-patient unit from the EDIS, by year. EDIS, Royal Free Hospital Eating Disorder Intensive Service.

In the original group, 29 EDIS patients were transferred to a specialist eating disorder unit (33.72%) over the 3 years. In the intensive family model group. 11 patients were transferred to an in-patient service (10.58%) over the 3 years. Figure 2 highlights the number of patients admitted to a specialist eating in the past 6 years.

A chi-squared test was performed to test whether the number of patients transferred to an in-patient service between groups differed significantly. The test showed a significant difference in the number of patients that were transferred to in-patient services with a small to medium effect size, as shown in Table 3 (*χ*^2^(1) = 6.03, *P* < 0.05, Cramer's *V* = 0.2).

### %mBMI

The mean %mBMI on admission to EDIS was 79.73 (s.d. = 8.91) in the traditional model before the pandemic and 80.89 in the intensive family model (s.d. = 8.77), as demonstrated in Table 2. The mean %mBMI on discharge from EDIS was 88.37 (s.d. = 9.82) for the traditional model group and 89.08 (s.d. = 7.68) for the intensive family model group. The difference in %mBMI from admission to discharge was also calculated. As Table 2 shows, the mean difference in %mBMI was 8.81 (s.d. = 10.09) for the traditional model group and 8.19 (s.d. = 7.50) for the intensive family model group.

An independent samples *t*-test was performed to test whether the change in %mBMI from admission to discharge differed significantly between groups, but this failed to reach significance (*t*(157) = 0.444, *P* = 0.658), as demonstrated in Table 3.

### Readmissions

There were eight readmissions to the intensive service for each group. In the traditional model group, seven of the eight readmissions were transferred to an in-patient service during at least one of their admissions, with four patients being transferred to in-patient services after both admissions to EDIS. The average time between admissions was 226.62 days (s.d. = 131.84).

In the intensive family model group, out of the eight readmissions, only one patient was transferred to an in-patient service after their first admission and was transferred to out-patient services after their second admission. The average time between admissions was 92.22 days (s.d. = 74.51).

## Discussion

Transforming our adolescent intensive eating disorder service from a group-based programme to an intensive family programme appears to have positively influenced the programme's outcomes. Since the modification of our programme approach, the length of admission to the intensive service has significantly lowered, and significantly fewer patients have been transferred to specialist in-patient services. The findings are consistent with previous research, where day programmes for adolescent eating disorders were restructured to be informed by family-based treatment.^11^ Moreover, the findings are consistent with the broader research showing that community intensive treatment for restrictive eating disorders can prevent hospital admission and/or in-patient admission to a specialist eating disorder unit.^5–9^

Length of admission to the EDIS significantly decreased since the change in our treatment approach. The length of admission varies across studies, but generally, the average length of admission appears to be 12 weeks (Simic et al,^2^ 12.8 weeks; Ornstein et al,^9^ 11.94 weeks; Ngo and Isserlin,^12^ 81.9 days), which is comparable to the mean length of treatment in the EDIS in the past 3 years. It is important to note that a key design feature of our programme is that it is integrated within a comprehensive out-patient service. Therefore, full-time attendance at the EDIS tapers down as the young person begins to make progress in their recovery, and the transition back to out-patient is gradual.

The rates of in-patient admissions from previous studies vary; from 35.7% in Australia,^6^ to 17% in the USA^9^ and 18% in the UK.^2^ In the past 3 years since the change of our approach, the number of transfers to specialist eating disorder units from the EDIS has significantly reduced (from 33.78 to 10.58%). Moreover, the number of patients being admitted to specialist eating disorder units from the EDIS is decreasing year on year, with only two patients being admitted in 2022–2023 compared with 12 patients in 2018–2019. This highlights the possibility that modifying our approach may have led to significantly fewer patients being admitted to costly specialist eating disorder units. Furthermore, all patients that were readmitted to the EDIS in the traditional model had at least one in-patient transfer, compared with just one patient in the intensive family model. This possibly highlights that more complex patients can be treated effectively in the community with the intensive family model, and may not have responded well to the group-based environment.

Group-based therapy may lead to the development of unhelpful alliances and competition between young people. This competitiveness, paired with perfectionism and low self-esteem among young people in the group, could exacerbate their eating disorder and jeopardise their recovery.^13^ Clinical observations, along with the preliminary findings of this paper, may echo these previous results and explain why the modification in our service approach improved the programme outcomes. However, a qualitative study where adolescents reviewed their experiences of an eating disorder day programme found that, although comparisons and comments from other patients could have a negative impact, having a group aspect to the programme was an overall positive experience because it gave an opportunity to connect with people with similar experiences, reduced isolation and increased motivation.^14^ Further studies are needed to examine the efficacy of group-based programmes compared with individualised family-based programmes.

There are alterative explanations of why the modified EDIS model appears to have improved the intensive treatment programme. It is likely that staff confidence and expertise has continued to develop as the programme evolves and expands. The new programme has likely had a significant positive effect on parents. Offering intensive family support has likely led to parents having greater self-efficacy in caring for their child with an eating disorder, and this should be measured in future research. Having the ability to now facilitate nasogastric feeding on site is a huge addition to the programme, and has likely had a significant impact on its efficacy. This helps to avoid paediatric ward admissions, where staff may not be as knowledgeable about eating disorders. The rise of referrals to eating disorder services following the COVID-19 pandemic likely had an impact on the availability of costly specialist eating disorder unit placements, which potentially affected the number of patients transferred to these services because of the possible unavailability of these placements.

### Strengths and limitations

The strengths of this study are the systematic analysis of real-life data, and not having exclusion criteria. There are several limitations to take into consideration when interpreting the results of this observational study. First, the data was collected retrospectively and additional information to characterise the sample and outcomes were not available, therefore limiting the scope of the results. Patient-rated outcome measures were not routinely collected previously, but should be included in further studies. Additional variables could be used to demonstrate how differences in diagnosis and severity can affect treatment outcomes. Clinical observations and previous findings have shown that family dysfunction, changes and disharmony may have a significant impact on eating disorder pathology and complexity,^15^ and this should be included in future research. It was not in the scope of this study to include and measure information on the use of nasogastric feeding; however, this would be an important aspect to consider in future research. Patient involvement and feedback was not within the scope of this study, but should also be acknowledged and considered for future research.

There are a significant amount of data missing from the %mBMI variable, which affected the sample size. This was a result of inconsistencies in the data being recorded and a change in patient data recording systems. However, it is important to note that %mBMI alone is not a valid predictor of treatment outcome in restrictive eating disorders, because of the use of nasogastric tube feeding. Despite the limitations, there are several strengths to the study, such as a relatively large sample size and the real-life clinical outcomes, thus improving the ecological validity of the results.

### Implications for clinical practice

Further longitudinal research is needed to examine the clinical and cost-effectiveness of intensive eating disorder community programmes. Service evaluations can help policy makers and other stakeholders enhance the quality of adolescent eating disorder intensive treatment. An evidence-based agreement is needed on the most important elements of intensive treatment to improve the quality and standardisation of care, especially as intensive services for adolescent eating disorders continue to be set up within the National Health Service. These results show that this may include the removal of group-based therapy to focus solely on intensive family treatment. This will ensure that we are fostering treatment programmes that are the most helpful in improving the outcomes of adolescent restrictive eating disorders.

## About the authors

**Ellen Hayes** is a senior assistant psychologist at the Specialist Child and Adolescent Eating Disorder Service, Royal Free Hospital, London, UK. **Nicola Tweedy** is an assistant psychologist at the Specialist Child and Adolescent Eating Disorder Service, Royal Free Hospital, London, UK. **Victoria Chapman** is the Clinical Lead and a consultant child and adolescent psychiatrist at the Specialist Child and Adolescent Eating Disorder Service, Royal Free Hospital, London, UK.

## Data Availability

Data and materials that support these findings are available from the corresponding author, E.H., upon reasonable request.
